# Comment on “Use of Carnosine for Oxidative Stress Reduction in Different Pathologies”

**DOI:** 10.1155/2016/5250386

**Published:** 2016-07-17

**Authors:** S. V. Jargin

**Affiliations:** Department of Public Health, Peoples' Friendship University of Russia, Clementovski per 6-82, Moscow 115184, Russia

I congratulate Prokopieva et al. for their review “Use of Carnosine for Oxidative Stress Reduction in Different Pathologies” [1]. The authors overview properties and biological effects of the antioxidant carnosine (Cn). Data on the use of Cn in various conditions are discussed. Special attention is given to the use of carnosine in neurologic, mental diseases and alcoholism. I have read the paper with a great interest. However, a comment seems to be necessary to formulate a praxis-relevant and evidence-based conclusion.

Cn or *β*-alanyl-L-histidine, an endogenously synthesized dipeptide, was reported to possess antioxidant properties [1, 2]. Cn enters blood being absorbed in the small intestine upon ingestion [2]. Cn is also synthesized in the human body; carnosine synthase is present in skeletal and heart muscle and certain brain regions and in other tissues. The synthesis rate of Cn appears to be predominantly rate-limited by the availability of *β*-alanine. Another precursor, L-histidine, is normally present in blood in sufficient concentrations [3]. The supply of *β*-alanine is dependent both on the hepatic synthesis from uracil and on the diet [2]. Cn penetrates through blood-brain barrier and is generally characterized by high bioavailability [1].

To decide whether supplementation of a substance is indicated, the question should be answered whether there can be a deficiency of that substance and if yes, whether it can be compensated for by a diet modification. Such deficiency appears to be improbable for Cn, which is abundant in muscles and can be synthesized in the body. Tissue Cn concentrations are influenced by the diet and are lower in vegetarians than in the general population [2, 4]. In vegetarians, muscle Cn content is limited by hepatic synthesis of *β*-alanine, whereas in omnivores there is additional dietary supply [5].

Cn was reported to be of importance for the antioxidant lens protection and preservation of its transparency, for prevention of certain neurologic and mental diseases. These potencies have been ascribed to the antioxidant properties of Cn [1, 2, 6, 7]. Among other potential applications, Cn eye drops have been recommended for cataracts [1, 6, 7]. However, if Cn concentration in body fluids is of importance, the incidence of corresponding conditions in vegetarians or population groups consuming less meat would be higher than average, which, to the best of our knowledge, has never been reported. Antioxidants affecting reactive oxygen species may have both harmful and beneficial effects [8]. The same is true particularly for Cn [9]. Generation of reactive oxygen species is a normal phenomenon in the course of aerobic metabolism [8]. Free radicals are not invariably toxic; some of them are necessary for the proper physiological functioning [10]. The redox status is maintained in equilibrium under the influence of many factors [11, 12]. The artificial support of the antioxidant status is considered to be not necessarily beneficial [11]. The topic of antioxidants is further complicated by conflicts of interest. Some antioxidants are propagated as dietary supplements or inexpensive substitutes for evidence-based medications. There are many examples of marketed substances without satisfactorily proven effectiveness beyond the placebo effect [13–17]. Publications of questionable reliability are sometimes used for advertising of drugs and dietary supplements, for their official registration, and for obtaining permissions for practical use. As a result, substances with unproven effects can be offered to the patients misinformed not only by advertising but also by some publications supposed to be scientific. The Cn eye drops sold in Russia are relatively expensive; they are prescribed to aged patients. In theory, Cn eye drops could be replaced by adequately prepared meat extract. Analogous suggestions have been made, for example, for osteoarthritis, to recommend for patients with low incomes to replace glycosaminoglycan-containing chondroprotectors with natural glycosaminoglycans that are abundant, for example, in animal cartilages and chicken wings [17]. To support the placebo effect, patients may be advised that the natural products can supply their organism with Cn similarly to drugs or dietary supplements. However, considering uncertainties about practical usefulness of antioxidants, discussed above, we would rather abstain from such recommendations. Even more precarious, because of complication risks [18], are recommendations of peribulbar injections of carcinine (an analog of Cn) as an antioxidant for prophylactic purposes [19]. Until recently, peribulbar injections of amino acid taurine (one of the most abundant amino acids in mammalian tissues [20, 21]) were used in the former SU as a preparation named Taufon in elderly patients for prophylactic purposes as well as for the treatment of macular dystrophy associated with atherosclerosis [22], while hematomas were observed as complications. Taufon has also been used as eye drops [23].

In conclusion, a stereotype that can be observed in some papers on normal metabolites such as Cn or taurine is as follows: metabolic importance of a substance is pointed out, which is true for many normal metabolites. In the second part of the paper, practical applications are discussed, although it remains unproven whether a deficiency of the substance ever occurs, and if it does, whether it can be compensated for by a diet, or a supplementation by drugs is really necessary. Note that drugs or dietary supplements might be more expensive for patients than a diet modification, for example, consumption of more meat products as a natural source of Cn or taurine. In any case, considering reported efficiency of Cn in chronic discirculatory encephalopathy, dementia, alcoholism, some mental disorders, and diabetes [1, 24, 25], an increase in consumption of meat products should be recommended for inhabitants of homes for the aged and psychiatric facilities. This seems to be the most important conclusion that can be derived from the article [1]. Benefits from the intake of Cn, other peptides, or amino acids in such patients may be seen as circumstantial evidence of malnutrition.
